# Dental Education

**Published:** 1884-07

**Authors:** 


### DENTAL EDUCATION.

---

An article appeared in the INDEPENDENT PRACTITIONER (June number) under the caption "Dental Education," by B. Merrill Hopkinson, D. D. S., of Baltimore, Md., professedly in the interest of a higher and purer standard of dental education, but mainly made up of a vicious attack on the Baltimore College of Dental Surgery. Is this article really what it professes to be, a *bona fide* effort to aid in the elevation of the standard of dental education?

We believe not, and further believe that we can show that it is not. The very tone and style of the article, from the point where the name of the Baltimore College of Dental Surgery is introduced into it, through the statement of cases, and down to the last line of the concluding paragraph, betray and express the feeling and spirit not of true friends of dental education, but of enemies of the Baltimore College of Dental Surgery.

This will become plain when we come to show whence this article emanated, and still more so when it is again read in the light of the facts which we will state, and we will state nothing as facts *of which we have not the best of proof ready for production* when the proper time comes.

Yet, while believing this, we will fearlessly meet the issue presented in it. While showing that the article containing this attack emanates from a rival institution, we will also show that the present Faculty of the Baltimore College of Dental Surgery has done nothing inconsistent with the custom and practice of the college, running back at least to 1869, fifteen years.

We know and can prove that the Faculty of the Baltimore College of Dental Surgery did, before the organization of the present Faculty, examine, and on being satisfied by such examination of their acquirements, proficiency and skill, grant in many cases diplomas to candidates who had not attended a course of lectures in that or any other dental college, and in one case, at least, in the same session in which Dr. Hopkinson graduated.

While we have the names of many of those in whose cases this was done in the last fifteen years, it would be manifestly indelicate and improper to bring them now before the public; they will be produced whenever it is proper to do so. Therefore, the ground taken in the article under consideration, that the alleged "irregularities" are of recent introduction and practiced only by the "present Faculty" of the Baltimore College of Dental Surgery, is entirely without foundation. But the present Faculty of the Baltimore College of Dental Surgery does not concede for a moment that such granting of diplomas, practiced by their predecessors and continued by them, is an irregularity, but contend that it is founded on sound principles and common sense. The position that they occupy on this point has been repeatedly set forth in the "Visitors' Letter," published annually in the catalogue. We quote the following paragraph from the "Visitors' Letter," as published in the catalogue of 1882-3. "Attention is again called to some of the prominent and distinguishing features of the regime of this institution, viz: 1st. The practical recognition of the principle that merit and proficiency, as developed by a thorough examination, should constitute the title to a diploma. 2d. A board of visitors, selected from among the prominent members of the profession in different parts of the country, and acting with the Faculty in an auxiliary and advisory capacity. And, 3d. Open examinations, at which the board is represented by a committee, said examinations being based on a series of written or printed questions, prepared by each professor, and embracing the whole course of lectures and instructions in his department."

The correctness of the principle of the first feature is acknowledged by many of the best men of the profession. The objections to the application of this principle to practice are not founded on the unsoundness of it, but on its liability (and in the view of some), its certainty to be abused.

The State Dental Societies practice on this principle, in that they, under authority of a law enacted by the legislatures of their respective States, at their request and through their influence, do by the agency of a board of dental examiners, appointed in most cases by themselves, examine all applicants and grant to such as are found qualified a license to practice, or a degree, without reference to where, from whom, or in what length of time they acquired their qualifications.

It is in accordance with this principle that so many dental educational institutions, while requiring attendance on two full courses of lectures, accept in lieu of one course five years' dental practice, including regular pupilage; that is, the matriculant having acquired elsewhere sufficient knowledge to entitle him to enter as a second course student, is not required to spend a whole session in the college to learn what he already knows.

Why not apply this same principle to one desiring a diploma, who knows enough of dentistry, theoretical and practical, to stand a first-class examination with a graduating class, and why compel him to attend a whole course of lectures to learn what he already knows? Besides, what properly conducted dental school has ever granted a diploma for attendance on two or more courses of lectures, without an examination of the applicant for it? Then, after all, is not a "satisfactory examination" really and in fact the only criterion by which the qualification of the candidate is measured?

These questions and positions, and probably others, will be proposed and discussed at the meeting of the Dental Faculties in New York City, in August next.

The Faculty of the College decided last year that they would, at the close of the following session, suspend the long-continued practice of the school in this respect, without, however, yielding their convictions of the soundness of their position. This was done from a due respect to the opinions and convictions of others, in a spirit of conciliation and with the expectation of being governed, after these points had been fully discussed, by the decision of the majority.

But now to the gist and gravamen of the article of B. Merrill Hopkinson, D. D. S., published in the June number of the INDEPENDENT PRACTITIONER. By the way, is this the same B. M. Hopkinson whose name lies before us, signed in a "round robin" with a number of other members of the graduating class of 1879–80, and attached to a paper condemning as a teacher the present dean of the Baltimore College of Dental Surgery, Professor R. B. Winder? Verily, round robins, like anonymous letters, are not brave things, and students are not competent judges of the qualifications of teachers.

On page 294, Dr. Hopkinson says: "I will now proceed to the statement of a few facts." So will we, and we will add some important ones omitted by him, and also prove some of *his* facts to be entirely untrue.

It is a matter of fact as shown in the annual catalogue of the Dental Department of the University of Maryland, of which F. J. S. Gorgas is now dean, that B. Merrill Hopkinson is an officer in that department—an inferior officer, it is true, but nevertheless an officer, being Assistant Demonstrator of Operative Dentistry. We think that if the Assistant Demonstrator of Operative Dentistry in the Dental Department of the University of Maryland had been as fully informed (as is evident from his article he was of other things) of the practices of the dental members of the Faculty of his department for years, while they were professors in the Baltimore College of Dental Surgery, and extending up to and through the session of '82, in voting to confer the degree and grant the diploma of D. D. S. without attendance, or on mere nominal attendance on lectures, this article would hardly have been fathered and published by him.

It is so clear and plain an inference as almost to amount to a fact, that as Assistant Demonstrator he could not have come officially in possession of the "names, dates and localities" and alleged facts of the four cases described by him in his communication, but must have obtained his information from the dean, to whose knowledge alone these alleged facts should originally come, and from whom alone a knowledge of them could be derived. Here we have an attack on the Baltimore College of Dental Surgery by an officer, if not by the officers of the Dental Department of the University of Maryland, a rival dental school, in which the dean of the latter is held up to public view as being rigidly virtuous and the Faculty of the former as being the loosest kind of characters. We should not need to go farther than this for the real motive and true animus of the article to which we are replying.

We have given above some of the facts omitted in that article, and will now give some more, and further on still more, which will form very interesting reading to all directly or remotely concerned. Before taking up separately the four cases mentioned, we will say that all the gentlemen named, except the two from Massachusetts, brought, in compliance with the requirements of the college, satisfactory evidence that they had been practicing dentistry more than ten years, and that they had in that time made a fair reputation for themselves in the profession.

All six of these gentlemen were examined by each professor in his department (as all other candidates are), and each professor voted on the result of that examination. The committee of the Board of Visitors were present, as they always are at all of these examinations, and at the Faculty meeting at which the vote on all candidates for graduation was taken. This is the regular practice every year, and, taken in connection with the quoted paragraph of the Visitors' Letter and the known practice of the college in this respect, should be evidence that the Faculty of the Baltimore College of Dental Surgery have nothing to conceal, but hold all their official acts and deeds open to the broad light of professional inspection. We will now take the last case (No. 4) first. The *alleged* facts in that case are that "Mr. C. S. W. Baldwin applied to the dean of the Dental Department of the University of Maryland on the last day of February, 1884, for admission to the graduating class, was quickly refused, and, *mirabile dictu*, was made a Doctor on March 6, 1884, by the Faculty of the Baltimore College of Dental Surgery."

The *real* facts are as follows:

The gentleman named wrote to the dean of the Dental Department of the University of Maryland as to terms of graduation in his case, and received the following reply, of which we have the original in the handwriting of, and signed by, F. J. S. Gorgas.

BALTIMORE, February 7, 1884.

DR. C. S. W. BALDWIN:

*Dear Sir*—Your letter is at hand, and its contents carefully noted. As three other members of the New Jersey State Dental Society, of eighteen and twenty years practice, desire a diploma from the University of Maryland Dental Department, the following arrangements have been made in their cases and accepted, and the same will be made to you: To come, on or about the twentieth of the present month and remain several days, matriculating, and during their stay dissecting the upper extremity, then return home, and next session pass the month of November at the University, a few days of January, and the greater part of the month of February, and be present during examination week (1st of March) and on commencement day, about the 14th or 15th of March. Such an arrangement will enable you to dissect this session, and the amount of fees to be paid this session will be five dollars for matriculation, and ten dollars for dissecting ticket. Next session the fees will be one hundred and five dollars, and diploma fee thirty dollars.

Such attendance will comply with the requirements of the University. Less time spent here would not do so, and besides, the State Associations are taking decided action against the conferring of diplomas by colleges without attendance, and some will, no doubt, during the coming year refuse to recognize diplomas which are bought.

I trust the arrangement I have described may suit you as it has the Secretary and two prominent members of your State Society, who are going to follow it out. I shall be much pleased to receive the specimens you refer to in your letter, for our museum.

Yours truly,

F. J. S. GORGAS, Dean.

In answer to further inquiries, he received the following, also in the handwriting of F. J. S. Gorgas, and signed by him:

BALTIMORE, February 15, 1884.

DR. C. S. W. BALDWIN:

*Dear Sir*—Our regular sessions end March 15th of each year, and the summer sessions are devoted altogether to practical work; hence, attendance on the latter is not regarded as equivalent to that at a regular session.

I should like to oblige you in every way possible, but the rules of the University are such that I cannot offer more than in my first letter. The Board of Regents are very particular in regard to the observance of all requirements. Besides, the State Board of Examiners are noting irregularities on the part of dental institutions, and will, through great pressure brought to bear during the coming summer, declare the diplomas of all institutions who graduate on a slight attendance, of no account. Pennsylvania may take this action soon in regard to a dental college in Baltimore, whose diploma is becoming worthless on account of irregularity and the mere selling of degrees.

Yours truly,

F. J. S. GORGAS.

The dates and contents of these documents prove the untruthfulness of the statement of the case by the author or authors of the article, and also prove the insincerity of the article, ostensibly from the pen of a disinterested party, but really of a demonstrator of operative dentistry in a dental school which is a rival of the Baltimore College of Dental Surgery, and whose dean agrees, in writing, to commit the irregularity of accepting two months or less presence at his school, for attendance on a full course of lectures which commences on the 1st of October, and ends in March, five full months.

The further real facts in this case are that Dr. C. S. W. Baldwin (for he has this title in the above letters) matriculated in the Baltimore College of Dental Surgery on the 22d or 23d day of February, 1884, having brought the required evidence of having been in practice ten years, and the endorsement of three dentists of high standing—one in New York City, one in Newark, N. J., and one in Montclair, N. J., as to the good standing and reputation he had acquired—underwent his examinations, as all other candidates for graduation did during five days, from February 26th to March 1st, inclusive, was found qualified, and received his diploma on March 6th.

We will not leave this case without giving the following statement which we have from Dr. Baldwin, and to which he is ready to testify on oath. He has never even seen the dean of the Dental Department University of Maryland, therefore he could not have applied to Dr. Gorgas, much less been refused by him, as stated in the article. He called to see Dr. Gorgas on the evening of February 19, 1884, but finding him not at home, was sent to Dr. Jas. H. Harris, the only other dental professor in their dental department. So disgusted was Dr. Baldwin with his disparagement of others that he decided to go to the old Baltimore College, and applied the next morning to its dean to matriculate, but could not do so until he had furnished the required endorsement.

We will next take the case No. 3. We have the sworn statement of Dr. T. H. Schaeffer, that he was looking for the Baltimore College of Dental Surgery, and by mistake called on Dr. Gorgas. That he was not refused, but after leaving him learned that he was no longer connected with the Baltimore College Dental Surgery, and, therefore, matriculated at that college, underwent a regular examination and received his diploma.

Case No. 2, that of Drs. J. F. Dowsley and F. A. Twitchell, is cited as one of irregularity, but is not so by any means. They were qualified to enter as second course students, as is shown by their matriculation with Dr. Gorgas, by virtue of having attended one course of lectures in a dental college. After being matriculated at the Dental Department of the University of Maryland, they passed a few days there, and all the rest of the session in attendance on lectures at the Baltimore College of Dental Surgery. They did not pay their "fees," as stated in the article, but only the matriculation fee of five dollars. By the way, is it not singular that of all the cases of graduates on examination by the Baltimore College of Dental Surgery, those only are mentioned in which the hundred and thirty dollars each slipped through the hands of the dean after he thought he had them safe? This, after all, would seem to be the true grievance, and to answer the question in the article, "why go further and speak specially of the other cases?"

The real facts of the remaing case (No. 1), as contained in a sworn statement of Drs. F. C. Barlow and C. A. Meeker, are as follows: On or about December 27th, 1883, Dr. Chas. A. Meeker was spoken to and told that he ought to go to Baltimore and graduate at the Dental Department of the University of Maryland at that session, and that the party so advising him would write a letter to the dean, and have him matriculated and graduated at that session, if he, Dr. Meeker, would send five dollars in the letter so written (of course he was to pay the other fees at the proper times).

The letter was written, the five dollars and a few lines from Dr. Meeker enclosed and mailed. In reply the dean wrote a letter (which we have) to Dr. Meeker, dated December 29th, 1883, acknowledging the receipt of the five dollars and enclosing a matriculation ticket. A few days after, Dr. F. C. Barlow was spoken to to a like effect, and he too was matriculated.

Both these gentlemen went to Baltimore about the 4th day of January, 1884. They called on Dr. Gorgas the next day, Saturday, and were given to understand by him that they could graduate at that session (which was what they had been told before leaving home); that Dr. Tiffany was very strict, but that he (the dean) had two votes and Dr. Harris one, and if they could get another one, these four votes would pass them even if Dr. Tiffany voted against them. They called on him again, at his request, the next day, Sunday, when he told them he had seen Dr. Tiffany about their graduating at that session, but could do nothing with him as he was opposed to it. They were then introduced to Dr. J. H. Harris, who, under the circumstances, broached the idea of their receiving an "honorary degree" at the end of that session.

They went home, and about January 25th, 1884, were informed that the medical members of the faculty would not consent to the granting of an honorary degree in their cases, so that they must come to the University at the next session, that their attendance on the lectures would be merely nominal, but that they would be examined at the end of the session.

On the 25th of February they called on the Demonstrator of Anatomy, procured dissecting tickets, were furnished a subject and went to dissecting a head. After they had finished dissecting, and just before leaving, they were told by the dean that they need only come two or three times at the next session, as it might suit their convenience, but it was necessary that they should be *seen* at the University, that their examination would be light, and they might rest assured of their diplomas, because he would guarantee them the votes of himself and Dr. Harris.

On reflecting and consulting on this, they concluded that a diploma obtained on these conditions would not possess the value that it should have, so they matriculated at the Baltimore College of Dental Surgery, underwent their examinations by all the professors, were pronounced qualified, and received their diplomas.

The slur on these gentlemen, on page 296 of the article, was uncalled for and without justification. They were among the original members who formed the State society, and have long held important positions in New Jersey, both having been President of the society—one for seven years its Secretary, and the other about the same length of time a member of the Board of Examiners.

One more fact, and we will close this reply. We have in our possession a document, of which the following is a true copy, except that for reasons before stated, we withhold the name on the same conditions:

"On or about the 14th of November, 1883, I visited the Dental Department of the University of Maryland. Met Dr. Jas. Harris. He stated to me that he knew my ability, and if I would matriculate at the college and stay awhile, I could go home and attend to my practice until near the commencement, and then return to the college and graduate, and that he would guarantee me a diploma. I told him that I was the beneficiary student to the old Baltimore College of Dental Surgery, and Dr. Harris said he was sure Dr. Gorgas would take my ticket and admit me as beneficiary student. Dr. Harris also stated that the old Baltimore College of Dental Surgery was defunct, and the present term would be the last of it."

"May 28th, 1884." ———

"Testified to before me this May 28th, 1884,

H. B. Gidelins, J. P."

We will conclude this reply by saying that the members of the "present faculty" of the Baltimore College of Dental Surgery have always been and are now ready to co-operate in any judicious, well-considered, and practical measure to raise the standard of graduation in the dental schools.

James B. Hodgkin, D. D. S.
Richard B. Winder, M. D., D. D. S.
M. Whilldin Foster, M. D., D. D. S.
J. H. Coyle, D. D. S.
Thomas S. Latimer, M. D.
James E. Lindsay, M. D.

*Governing Faculty of the Baltimore College of Dental Surgery.*

